# The Nab2 RNA-binding protein patterns dendritic and axonal projections through a planar cell polarity-sensitive mechanism

**DOI:** 10.1093/g3journal/jkac100

**Published:** 2022-04-26

**Authors:** Edwin B Corgiat, Sara M List, J Christopher Rounds, Dehong Yu, Ping Chen, Anita H Corbett, Kenneth H Moberg

**Affiliations:** 1 Department of Cell Biology, Emory University School of Medicine, Emory University, Atlanta, GA 30322, USA; 2 Department of Biology, Emory University, Atlanta, GA 30322, USA; 3 Genetics and Molecular Biology Graduate Program, Emory University, Atlanta, GA 30322, USA; 4 Neuroscience Graduate Program, Emory University, Atlanta, GA 30322, USA

**Keywords:** Nab2, RNA-binding protein, planar cell polarity, mushroom body, axon, ddaC neuron, dendrite, intellectual disability

## Abstract

RNA-binding proteins support neurodevelopment by modulating numerous steps in post-transcriptional regulation, including splicing, export, translation, and turnover of mRNAs that can traffic into axons and dendrites. One such RNA-binding protein is ZC3H14, which is lost in an inherited intellectual disability. The *Drosophila melanogaster* ZC3H14 ortholog, Nab2, localizes to neuronal nuclei and cytoplasmic ribonucleoprotein granules and is required for olfactory memory and proper axon projection into brain mushroom bodies. Nab2 can act as a translational repressor in conjunction with the Fragile-X mental retardation protein homolog Fmr1 and shares target RNAs with the Fmr1-interacting RNA-binding protein Ataxin-2. However, neuronal signaling pathways regulated by Nab2 and their potential roles outside of mushroom body axons remain undefined. Here, we present an analysis of a brain proteomic dataset that indicates that multiple planar cell polarity proteins are affected by Nab2 loss, and couple this with genetic data that demonstrate that Nab2 has a previously unappreciated role in restricting the growth and branching of dendrites that elaborate from larval body-wall sensory neurons. Further analysis confirms that Nab2 loss sensitizes sensory dendrites to the genetic dose of planar cell polarity components and that Nab2-planar cell polarity genetic interactions are also observed during Nab2-dependent control of axon projection in the central nervous system mushroom bodies. Collectively, these data identify the conserved Nab2 RNA-binding protein as a likely component of post-transcriptional mechanisms that limit dendrite growth and branching in *Drosophila* sensory neurons and genetically link this role to the planar cell polarity pathway. Given that mammalian ZC3H14 localizes to dendritic spines and controls spine density in hippocampal neurons, these Nab2-planar cell polarity genetic data may highlight a conserved path through which Nab2/ZC3H14 loss affects morphogenesis of both axons and dendrites in diverse species.

## Introduction

While many key developmental events are triggered by extracellular factors that signal through cytoplasmic cascades to alter nuclear gene transcription, other key events are triggered by shifts in post-transcriptional processing or localization of mRNAs that guide cell fates and differentiation. Importantly, the fidelity of these mRNA-based developmental mechanisms relies on RNA-binding proteins (RBPs) that associate with nascent RNAs and regulate splicing, export, stability, localization, and translation (Schieweck *et al.* 2021). These key regulatory mechanisms are particularly evident in the developing nervous system, where mutations in genes encoding RBPs are often linked to human diseases. Examples of this linkage include Fragile-X mental retardation protein (Gross *et al.* 2012), the survival of motor neuron protein (Edens *et al.* 2015), and the TAR DNA-binding protein 43 (Agrawal *et al.* 2019; Gebauer *et al.* 2021). Sensitivity of the central and peripheral nervous systems to loss of RBPs has been attributed to the importance of post-transcriptional mechanisms, such as local mRNA translation and 3′-UTR extension (Mattioli *et al.* 2017; Thelen and Kye 2019; Engel *et al.* 2020), that enable fine-tuned spatiotemporal control of neuronal gene expression. This spatiotemporal control of mRNA processing and translation plays an important role in forming complex dendritic architectures and the uniquely polarized morphology of neurons (Lee *et al.* 2003). Accordingly, neurological diseases caused by mutations in genes encoding RBPs often include defects in axonal or dendritic morphology (Jung *et al.* 2012; Hornberg and Holt 2013; Holt *et al.* 2019), and in some cases, these axonal and dendritic defects can be traced to defective post-transcriptional control of one or a few mRNAs normally bound by the corresponding RBP.

The human *ZC3H14* gene encodes a ubiquitously expressed zinc-finger, polyadenosine RBP (ZnF CysCysCysHis #14) that is lost in an inherited form of intellectual disability (Pak *et al.* 2011). Studies in multiple model organisms have begun to define functions for ZC3H14 in guiding neuronal morphogenesis. Analysis of the sole *Drosophila* ZC3H14 homolog, Nab2, detects cell-autonomous requirements in Kenyon cells (KCs) for olfactory memory as well as axonal branching and projection into the brain mushroom bodies (MBs; Kelly *et al.* 2016; Bienkowski *et al.* 2017), twin neuropil structures that are the center for associative olfactory learning in insects (Thum and Gerber 2019). Significantly, transgenic expression of human ZC3H14 only in fly neurons is sufficient to rescue a variety of *Nab2* null phenotypes (Pak *et al.* 2011; Kelly *et al.*^2014^, ^2016^), supporting a model in which Nab2 and ZC3H14 share critical molecular roles and mRNA targets. The *Zc3h14* gene is not essential in mice but its loss results in defects in working memory (Rha *et al.* 2017) and dendritic spine morphology (Jones *et al.* 2021). An accompanying proteomic analysis of *Zc3h14* knockout hippocampi identified several proteins involved in synaptic development and function that change in abundance upon ZC3H14 loss (Rha *et al.* 2017) and are thus candidates to contribute to *Zc3h14* mutant phenotypes. Intriguingly, ZC3H14 localizes within dendritic spines in hippocampal neurons in culture and homologs of ZC3H14-regulated proteins in the mouse hippocampus are also sensitive to Nab2 loss in the developing *Drosophila* pupal brain (Corgiat *et al.* 2021), suggesting conserved links between Nab2 and ZC3H14 and neurodevelopmental pathways.

A variety of intercellular signaling mechanisms plays required roles in sensing extracellular cues that guide the complex axonal and dendritic structures that characterize specific areas of the central and peripheral nervous system (CNS and PNS). These cascades can respond to long-range directional cues, such as Netrin signaling, or to short-range directional cues from the Slit-Robo, Abl-Ena, and Semaphorin pathways (Puram and Bonni 2013; Stoeckli 2018). One pathway with an emerging role in both axonal and dendritic development is the planar cell polarity (PCP)-noncanonical Wnt pathway (Zou 2004; Andre *et al.* 2012; Zou 2012; Gombos *et al.* 2015; Misra *et al.* 2016). PCP signals are based on the asymmetric distribution of 2 apically localized transmembrane complexes, which in *Drosophila* correspond to the Stan-Vang-Pk complex (Starry Night aka Flamingo–Van Gogh–Prickle) and the Stan-Fz-Dsh-Dgo complex (Frizzled–Disheveled–Diego); these complexes are intracellularly antagonistic but intercellularly attractive, leading to apical polarization across an epithelial plane (Taylor *et al.* 1998; Boutros and Mlodzik 1999; Vladar *et al.* 2009; Goodrich and Strutt 2011; Adler 2012; Peng and Axelrod 2012; Adler and Wallingford 2017; Mlodzik 2020). Core PCP components signal to downstream effector molecules that exert localized effects on the F-actin cytoskeleton (Courbard *et al.* 2009; Adler 2012; Soldano *et al.* 2013; Fagan *et al.* 2014; Gombos *et al.* 2015), which in turn guides epithelial traits like proximal-distal wing hair orientation in *Drosophila* and sensory hair cell polarity in the mouse cochlea (Jones and Chen 2007; Qian *et al.* 2007; Simons and Mlodzik 2008; Chacon-Heszele and Chen 2009; Rida and Chen 2009; Alpatov *et al.* 2014). One such factor is encoded by the *β amyloid protein precursor-like* (*Appl*) gene and modulates the PCP pathway during axonal and dendritic outgrowth (Soldano *et al.* 2013; Liu *et al.* 2021). Importantly, PCP is required for axon guidance in specific groups of neurons in *Drosophila*, *C*aenorhabditis *elegans*, mice, and chick, and for dendritic branching of mouse cortical and hippocampal neurons, and *Drosophila* body-wall sensory neurons (Hindges *et al.* 2002; McLaughlin and O’Leary 2005; Schmitt *et al.* 2006; Shafer *et al.* 2011; Cang and Feldheim 2013; Yoshioka *et al.* 2013; Hagiwara *et al.* 2014; Yasumura *et al.* 2021). For example, loss of the murine *Vang* homolog *Vangl2* leads to defects in axon guidance of spinal cord commissural axons (Shafer *et al.* 2011), and *dsh* mutants in *C. elegans* cause neuronal projection and morphology defects (Zheng *et al.* 2015). In *Drosophila*, loss of the core PCP components *stan*, *Vang*, *pk*, *fz*, or *dsh* individually disrupt α and β axon projection into the MBs (Shimizu *et al.* 2011; Ng 2012). Intriguingly, loss of *stan* or its LIM (LIN-11, Isl-1 and MEC-3)-domain adaptor *espinas* (*esn*) also disrupts dendritic self-avoidance among the class IV dendritic arborization (da) neurons (Matsubara *et al.* 2011), demonstrating a requirement for PCP proteins in both axon and dendrite morphogenesis within sets of neurons in the CNS and PNS.

Integrating data from 2 of our recent studies provide evidence for pathways through which the Nab2 RBP could guide axonal and dendritic projections. These analyses, one a genetic modifier screen based on a *GMR-Nab2* rough eye phenotype (Lee *et al.* 2020) and the other a proteomic analysis of *Nab2* null pupal brains (Corgiat *et al.* 2021), each suggests a link between Nab2 and the PCP pathway. The *GMR-Nab2* modifier screen identified alleles of PCP components, both core components and downstream effectors (e.g. *Vang*, *dsh*, *fz*, *stan*, *pk*, *Appl*, and the formin *DAAM*), as dominant modifiers of *Nab2* overexpression phenotypes in the retinal field (Lee *et al.* 2020). In parallel, gene ontology (GO) analysis of proteomic changes in *Nab2* null brains detected enrichment for dendrite guidance and axodendritic transport GO terms among affected proteins (Corgiat *et al.* 2021), which include the core PCP factor Vang and the PCP accessory factor A-kinase anchor protein 200 (Akap200). Significantly, *Drosophila* Vang and its murine homolog Vangl2 are one of 6 pairs of homologs whose levels change significantly in *Nab2* null fly brains and *Zc3h14* knockout mouse hippocampi (Rha *et al.* 2017; Corgiat *et al.* 2021), suggesting a conserved relationship between Nab2/ZC3H14 and the PCP pathway in the metazoan CNS.

Considering observations outlined above, we have investigated interactions between *Nab2* and PCP genes in 2 neuronal contexts—CNS axons of the *Drosophila* pupal MB α- and β-lobes, and in larval dendrites of class IV dorsal dendritic arbor C (ddaC) neurons—which provide complementary settings to analyze the Nab2-PCP link in axonal and dendritic compartments. We detect enrichment for PCP proteins among brain-enriched proteins affected by Nab2 loss, and a pattern of genetic interactions between *Nab2* and multiple PCP alleles in both MB axons and ddaC dendrites that are consistent with Nab2-regulating axon and dendrite outgrowth by PCP-linked mechanisms. However, differences in how individual PCP alleles modify axonal vs dendritic *Nab2* mutant phenotypes suggest that the Nab2-PCP relationship may depend on cellular context (i.e. pupal KCs vs larval ddaC neurons). Cell type-specific RNAi indicates that Nab2 acts cell autonomously to guide axon and dendrite growth, implying a potentially direct link between Nab2 and one or more PCP components within KCs and ddaC neurons. Collectively, these data demonstrate that Nab2 is required to regulate axonal and dendritic growth through a PCP-sensitive mechanism that has the potential to be conserved across species.

## Materials and methods

### Drosophila genetics

All crosses were maintained in humidified incubators at 25°C with 12 h light-dark cycles unless otherwise noted. The *Nab2^ex3^* loss-of-function mutant has been described previously (Pak *et al.* 2011). Alleles and transgenes: *Nab2^EP3716^* [referred to as “*Nab2 oe*”; Bloomington (BL) #17159], *UAS-Nab2^RNAi^* (Vienna *Drosophila* Research Center, #27487), *UAS-fz2^RNAi^* (BL #27568), *appl^d^* (BL #43632), *dsh^1^* (BL #5298), *Vang^stbm-6^* (BL #6918), *pk^pk-sple-13^* (BL #41790), *Vang^EGFP.C^* (“*Vang-eGFP*”) (gift of D. Strutt), *ppk-Gal4; UAS-mCD8::GFP* (gift of D. Cox), and *w^1118^* (“control”).

### *Drosophila* brain dissection, immunohistochemistry, visualization, and statistical analysis

Brain dissections were performed essentially as previously described (Kelly *et al.* 2016). Briefly, 48–72 h after puparium formation (APF) brains were dissected at 4°C in PBS (1× PBS), fixed in 4% paraformaldehyde at RT, washed 3× in PBS, and then permeabilized in 0.3% PBS-T (1× PBS, 0.3% TritonX-100). Following blocking for 1 h (0.1% PBS-T, 5% normal goat serum), brains were stained overnight in block+primary antibodies. After 5× washes in PBS-T (1× PBS, 0.3% TritonX-100), brains were incubated in block for 1 h, moved into block+secondary antibody for 3 h, then washed 5× in PBS-T and mounted in Vectashield (Vector Labs). Antibodies used: anti-FasII 1D4 (Developmental Studies Hybridoma Bank) at 1:50 dilution, anti-GFP polyclonal (ThermoFisher Catalog# A-11122) at a 1:200 dilution, and anti-nc82 (Developmental Studies Hybridoma Bank) at 1:50 dilution. Whole-brain images were captured on a Nikon AR1 HD25 confocal microscope using NIS-Elements C Imaging software v5.20.01, and maximum intensity projections were generated in ImageJ Fiji. Mushroom body morphological defects were scored as α-lobe thinning/missing and β-lobe fusion/missing for *control*, *Nab2^ex3^*, and PCP alleles (e.g. *Vang^stbm-6^/+*, *appl^d^/+*, and *dsh^1^/+* paired with *control* or *Nab2^ex3^*). Statistical analyses for MB phenotypes and plotting performed using GraphPad Prism8. Significance is determined using Student’s *t*-test or ANOVA as indicated in figure legends. Error bars representing standard deviation. Significance scores indicated are *P ≤ 0.05, **P ≤ 0.01, and ***P ≤ 0.001.

### *Drosophila* neuron live imaging confocal microscopy, neuronal reconstruction, data analyses, and statistical analysis

Live imaging of class IV ddaC neurons was performed essentially as described in Iyer *et al.* (2013) and Clark *et al.* (2018). Briefly, wandering stage third instar *ppk-Gal4, mCD8::GFP* labeled larvae were mounted in 1:5 (v/v) diethyl ether: halocarbon oil under an imaging bridge of two 22* *×* *22* *mm glass coverslips topped with a 22* *×* *50* *mm glass coverslip. ddaC images were captured on an Olympus FV 1000 BX61WI upright microscope using Olympus Fluoview software v4.2. Maximum intensity projections were generated with ImageJ Fiji. Neuronal reconstruction was performed with the TREES toolbox (Theisen *et al.* 1994). MathWorks Matlab R2010a v7.10.0.499 (Natick, MA) was used to process 2D stacks with local brightness thresholding, skeletonization, and sparsening to leave carrier points (Cuntz *et al.* 2010). Dendritic roots were defined at the soma and used to create synthetic dendritic arbors. Reconstruction parameters were equivalent across neurons. Various morphological metrics were obtained using the TREES toolbox including: Sholl analysis, total cable length, maximum path length, number of branch points, mean path/Euclidean distance, maximum branch order (maxbo), mean branch order (meanbo), mean branch angle, mean path length, field height/width, center of mass x, and center of mass y. These metrics were extracted in batch processing using in-house custom scripts and exported into RStudio v1.1.453 (Vienna, Austria), where quantification was visualized using other in-house custom scripts. Statistical analyses for ddaC phenotypes and plotting were performed using RStudio and Matlab. Balloon plots showing phenotypic data generated using either ddaC measurements generated in Matlab or MB defect counts. Balloon plots generated using RStudio v1.1.453 ggpubr v0.2 (Alboukadel 2018; R Development Core Team 2018).

### Global proteomics

MS/MS-LC data were previously described in Corgiat *et al.* (2021). Briefly, 10 biological replicates of 24 h apf control (*w^1118^*) or *Nab2^ex3^* pupal brains (60 brains per replicate) were lysed in urea buffer (8 M urea, 100 mM NaHPO4, pH 8.5) with HALT protease and phosphatase inhibitor (Pierce/Thermo Scientific) and processed at the Emory Proteomics Core. Separate samples were prepared for male and female brains. Label-free quantification analysis was adapted from a previously published procedure (Seyfried *et al.* 2017). Data were analyzed using MaxQuant v1.5.2.8 with Thermo Foundation 2.0 for RAW file reading capability. Spectra were searched using the search engine Andromeda and integrated into MaxQuant against the *Drosophila melanogaster* Uniprot database (43,836 target sequences). Analyses presented here used RStudio v1.1.453 (R Development Core Team 2018), custom in-house scripts, and the following packages: ggpubr v0.2 (Alboukadel 2018), cluster v2.1.0 (Maechler *et al.* 2016), and GOplot v1.0.2 (Walter *et al.* 2015), to examine “planar cell polarity” annotated proteins. GO analyses were performed using FlyEnrichr (FlyEnrichr: amp.pharm.mssm.edu/FlyEnrichr/; Chen *et al.* 2013; Kuleshov *et al.*^2016^, ^2019^), a *Drosophila* specific GO enrichment analysis package.

## Results

### Nab2 loss alters levels of PCP pathway proteins in the *Drosophila* brain

**Fig. 1.**
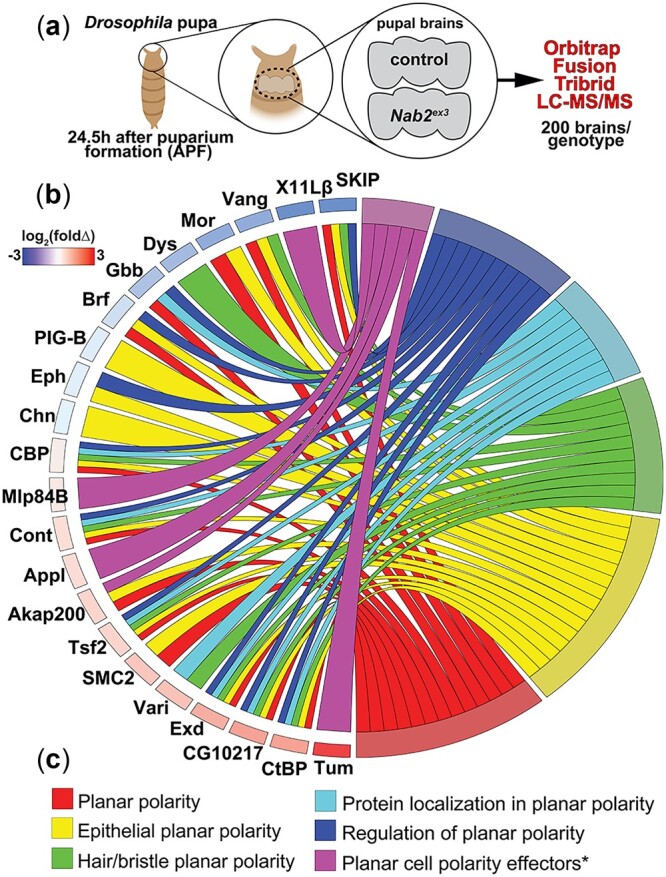
Nab2 loss alters levels of PCP pathway proteins in the *Drosophila* brain. a) Schematic summary of quantitative proteomic analysis of *Nab2^ex3^* pupal brains dissected from *control* or *Nab2^ex3^* pupa 24.5 h APF. Ten samples per genotype, each composed of 20 brains (i.e. 200 *control* brains and 200 *Nab2^ex3^* brains) were processed and analyzed using an Orbitrap Fusion Tribrid Mass Spectrometer and data were quantified using MaxQuant against the *D. melanogaster* Uniprot database. b) Chord plot analysis of protein abundance changes in *Nab2^ex3^* relative to *control* for selected color-coded PCP ontology terms. Heat map indicates fold-change in abundance of each protein (log_2_(*Nab2^ex3^*/*control*)).

Our recent study comparing proteomic changes in *Drosophila* pupal brains lacking Nab2 identified *PCP* GO terms as one category of significantly altered factors (Corgiat *et al.* 2021; Fig. 1a). A deeper analysis of this protein dataset detects enrichment for 5 PCP-related GO terms (*establishment of planar polarity*, *establishment of epithelial cell planar polarity*, *establishment of body hair or bristle planar polarity*, *protein localization involved in planar polarity*, and *regulation of establishment of planar polarity*; Fig. 1b) based on 17 proteins. This set of proteins includes the core PCP component Van Gogh (Vang) and 5 putative PCP effectors: the Tumbleweed GTPase activating protein (Sotillos and Campuzano 2000; Jones *et al.* 2010), the neuron-specific PCP modulator Appl (Singh and Mlodzik 2012; Soldano *et al.* 2013; Liu *et al.* 2021), the anchoring protein Akap200 (Jackson and Berg 2002; Weber *et al.* 2012; Bala Tannan *et al.* 2018), the endocytic regulator X11Lβ (Gross *et al.* 2013), and the muscle LIM-domain protein at 84B (Mlp84B; Weber *et al.* 2012). Together these factors represent 6.4% of the total differentially expressed proteins in *Nab2^ex3^* pupal brains relative to control (346 proteins in total; see Corgiat *et al.* 2021; Supplementary Table 1). The Vang protein (decreased by a factor of 5 in *Nab2^ex3^* vs control) and Appl protein (increased by a factor of 1.5 in *Nab2^ex3^* vs control) are particularly notable because alleles of these genes dominantly modify phenotypes produced by *GMR-Gal4* driven Nab2 overexpression in the developing retinal field (Lee *et al.* 2020).

### PCP components dominantly modify Nab2 axonal phenotypes

**Fig. 2.**
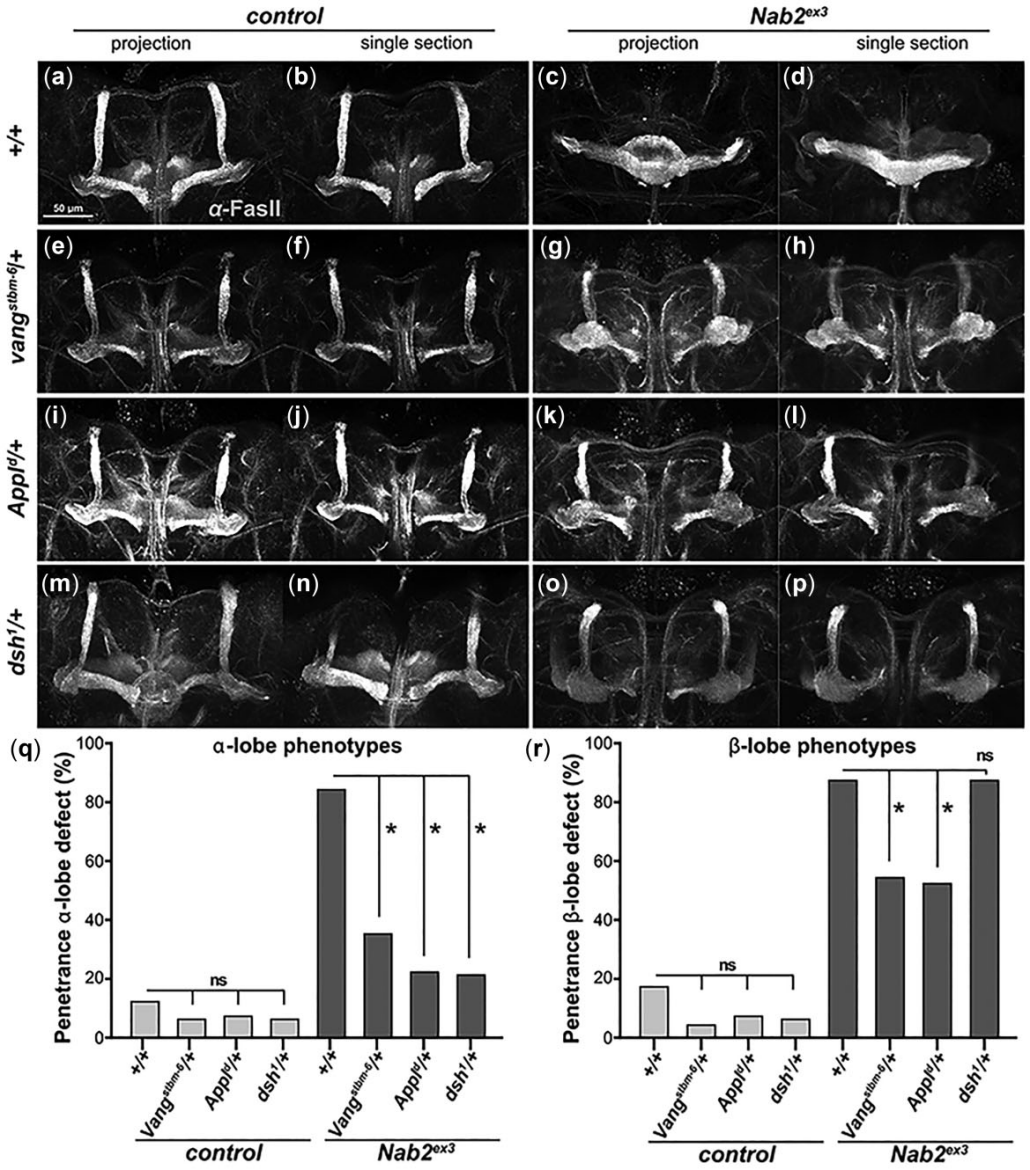
PCP components dominantly modify Nab2 axonal phenotypes. Paired maximum intensity Z-stack projections images and single transverse sections of anti-Fasciclin II (FasII) stained 48–72 h pupal brains from **(**a, b) *control* or (c, d) *Nab2^ex3^* animals, or each of these genotypes combined with (e–h) *Vang^stbm6^/+*, (i–l) *Appl^d^/+*, or (m–p) *dsh^1^/+*. Frequencies of (q) α-lobe or (r) β-lobe structure defects in these genotypes using the scoring system as described in Experimental Procedures. *Nab2^ex3^* brains show high penetrance thinning/loss of α-lobes (85%) and fusion/missing of β-lobe (88%) that are dominantly suppressed by *Vang^stbm-6^* (49% α-lobe and 33% β-lobe defects) and *Appl^d^* (62% α-lobe and 35% β-lobe defects). *dsh^1^* selectively suppresses *Nab2^ex3^* α-lobe defects to 63%. Significance is determined using Student’s *t*-test or ANOVA as indicated in figure legends. Significance scores indicated are *P ≤* *0.05, **P ≤* *0.01, and ***P ≤* *0.001.

To pursue the Nab2-PCP link in the developing CNS, we tested whether axon projection defects in MBs homozygous for the *Nab2^ex3^* null allele (Pak *et al.* 2011) are sensitive to subtle modulation of PCP pathway activity using single copies of loss-of-function alleles of PCP components. Our previous work established genetic interactions between Nab2 and an array of PCP/Wnt alleles in the adult *Drosophila* eye (Lee *et al.* 2020). Here, we focused on 3 of these factors: the core PCP/Wnt factor Vang, which proteomic data indicate is reduced by a factor of 5 in *Nab2^ex3^* brains (Corgiat *et al.* 2021), the accessory factor Appl (Amyloid precursor protein-like), which is a proposed PCP/Wnt coreceptor and has established links to neurological disease (Singh and Mlodzik 2012; Soldano *et al.* 2013; Liu *et al.* 2021), and the PCP/Wnt cytoplasmic adaptor Dsh, which also genetically interacts with *Nab2* in the wing to control hair polarity (Lee *et al.* 2020). As has been observed in *Nab2^ex3^* adult brains (Kelly *et al.* 2016; Bienkowski *et al.* 2017), *Nab2^ex3^* mutant pupal brain at 48–72 h APF display highly penetrant defects in structure of the α-lobes (85% thinned or missing) and β-lobes (88% fused or missing) as detected by anti-Fas2 staining (Fig. 2, a–d, q, and r). Both the *Vang^stbm6^* and *Appl^d^* loss-of-function alleles have no effect on MB structure in an otherwise wildtype background but suppress the frequency of *Nab2^ex3^* α-lobe defects from 85% to 49% in a *Vang^stbm6^/+* heterozygous background and to 62% in a *Appl^d^/+* heterozygous background; the frequency of *Nab2^ex3^* β-lobe defects drops from 88% to 33% in *Vang^stbm6^/+* heterozygous background and to 35% in *Appl^d^/+* heterozygous background (Fig. 2, e–f, i, j, m, and n). The PCP-specific allele *dsh^1^* (Theisen *et al.* 1994; Gombos *et al.* 2015) lowers *Nab2^ex3^* α-lobe defects from 85% to 63% but has no effect on the frequency or severity of *Nab2^ex3^* β-lobe defects (Fig. 2, q and r; Supplementary Fig. 1). Intriguingly, animals with single copies of *Vang^stbm6^*, *Appl^d^*, and *dsh^1^* in the *Nab2^ex3^* homozygous background also develop an MB phenotype not observed in any single mutant: a bulbous, Fas2-positive lobe located where the peduncle splits into the 5 lobes (α, α′, β, β′, γ; arrowhead in Fig. 2, g, k, and o). The basis of this bulbous phenotype is unclear but may indicate that lowering levels of PCP proteins in KCs that also lack Nab2 leads to a novel axon guidance defect among α/β axons. In sum, these data reveal a pattern of dose-sensitive genetic interactions between *Nab2* and PCP alleles that indicate that Nab2 loss sensitizes MB development to reduced PCP signaling.

### Nab2 is required to restrict dendritic branching and projection

**Fig. 3.**
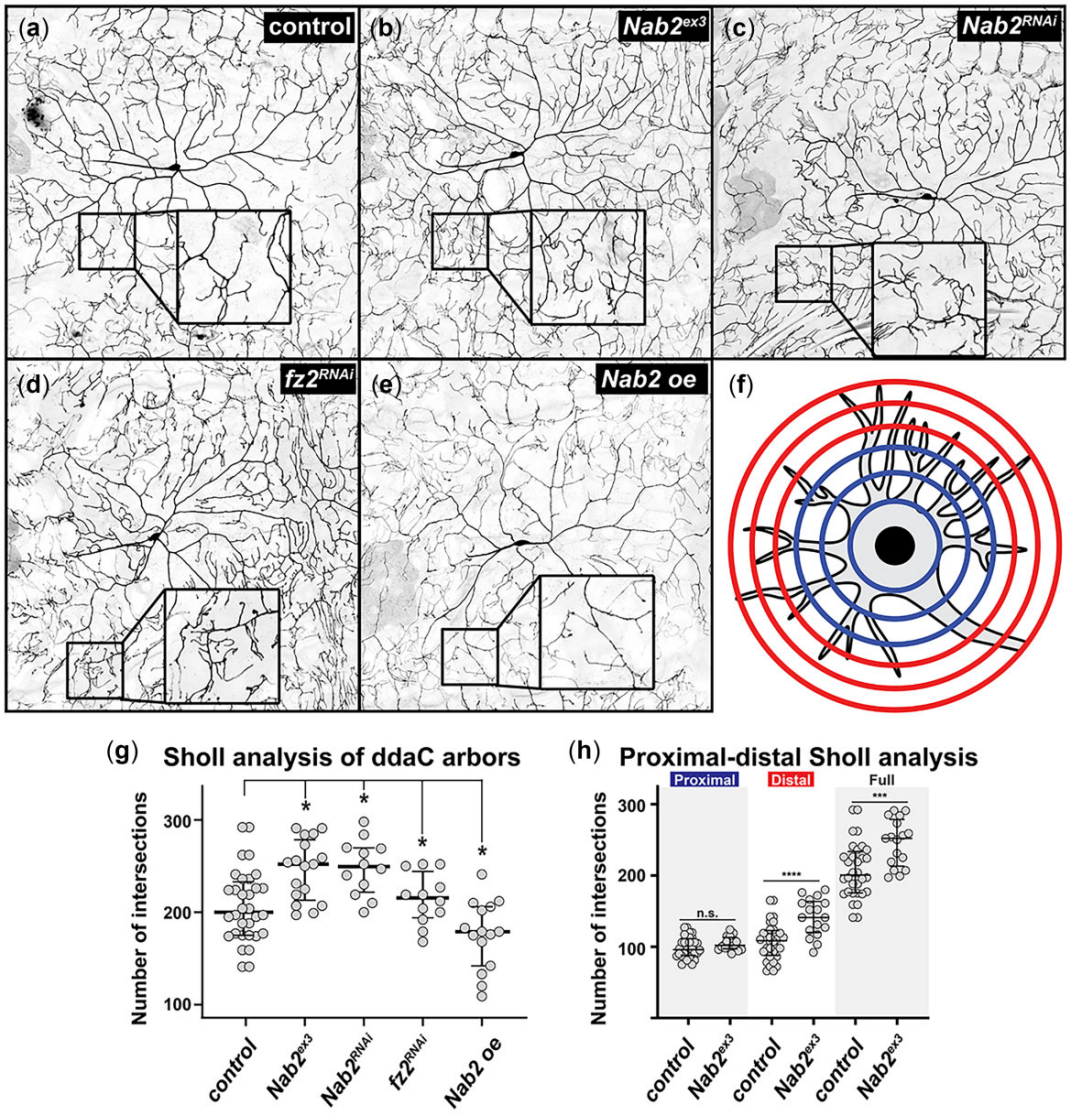
Nab2 is required for proper dendritic development. Inverted intensity images of *Drosophila* class IV ddaC neurons from (a) *pickpocket (ppk)-Gal4, UAS-GFP*, (b) *Nab2^ex3^*, (c) *ppk-Gal4, UAS-GFP, Nab2^RNAi^*, (d) *ppk-Gal4, UAS-GFP, fz2^RNAi^*, and (e) *ppk-Gal4, UAS-GFP, Nab2^oe^* L3 larvae. Inset black boxes show high magnification views of dendritic arbors*.* (f) Diagram depicting the concentric rings used to perform Sholl analysis overlaid on the dendritic arbor of a neuron. The half of the rings proximal to the soma labeled in blue; the half of the rings distal to the soma labeled in red. g, h) Quantification of branching complexity by Sholl analysis of total intersections across dendritic arbor; bars represent median and upper/lower quartile, * *P *<* *0.05. g) Sholl analysis of full dendritic arbor. Median Sholl intersection values are 200 in *ppk-Gal4, UAS-GFP* (*n *=* *32), 252 in *Nab2^ex3^* (*n *=* *17), 250 in *ppk-Gal4, UAS-GFP, Nab2^RNAi^* (*n *=* *12), 216 in *ppk-Gal4, UAS-fz^RNAi^* (*n *=* *12), and 179 in *ppk-Gal4, UAS-Nab2^oe^* (*n *=* *15). h) Sholl analysis of proximal and distal dendritic arbors. Median Sholl intersection values for *ppk-Gal4, UAS-GFP* (*n *=* *32) are 96 proximal and 108.5 distal, while median Sholl intersection values for *Nab2^ex3^* are 102 proximal and 141 distal. Significance is determined using Student’s *t*-test or ANOVA as indicated in figure legends. Error bars in (g) and (h) represent standard deviation. Significance scores indicated are *P ≤* *0.05, **P ≤* *0.01, and ***P ≤* *0.001.

Loss of murine *Zc3h14* causes defects in dendritic spine morphology among hippocampal neurons (Jones *et al.* 2021) prompted us to test whether Nab2–PCP interactions in axons are also conserved in developing dendrites. For this approach, we visualized dendrites of *Drosophila* class IV ddaC neurons located in the larval body wall using a *pickpocket (ppk)-Gal4, UAS-GFP* system and quantified branching using Sholl intersection analysis (Fig. 3f;Cuntz *et al.* 2010). In wandering stage L3 larvae, complete loss of Nab2 leads to increased dendritic branch complexity as measured by the number of Sholl intersections relative to control (median of 200 in *ppk>GFP* vs median of 252 in *Nab2^ex3^*; Fig. 3, a, b and, g), which is phenocopied by Nab2 RNAi depletion in ddaC neurons (median of 250 intersections in *ppk>Nab2^RNAi^*; Fig. 3, c and g). Nab2 overexpression in ddaC neurons using the *Nab2^EP3716^* transgene has the inverse effect of decreasing Sholl intersections (median of 179 in *ppk>Nab2*; Fig. 3, e and g). Significantly, RNAi depletion of the Wnt/PCP receptor *frizzled 2* in ddaC neurons also increases Sholl intersections (median of 216 in *ppk>fz2^RNAi^*; Fig. 3, d and g), confirming prior work that Wnt/PCP signaling is involved in ddaC dendritic development (Misra *et al.* 2016). Intriguingly, the increased Sholl intersections in *Nab2^ex3^* arbors are concentrated in distal segments (Fig. 3h), suggesting that the role of Nab2 in dendritic development becomes more significant with increasing distance from the cell soma.

**Fig. 4.**
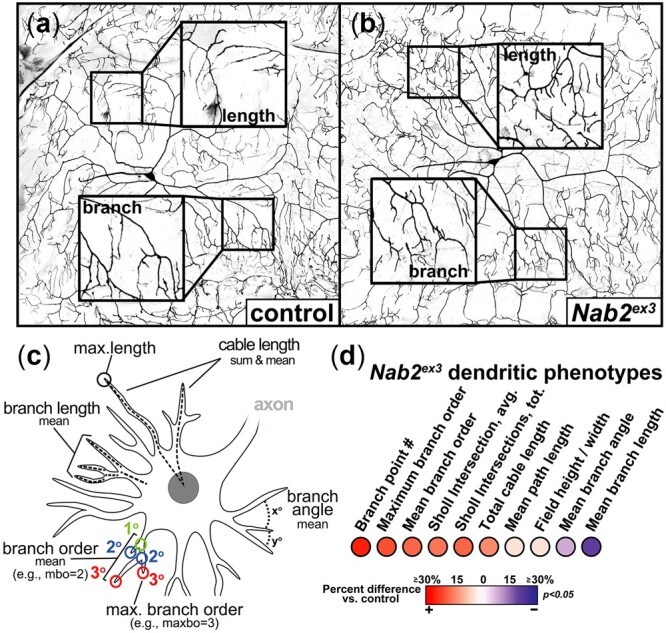
Nab2 restricts dendritic branching and projection. Inverted intensity images of *Drosophila* class IV ddaC neurons from (a) *control +/+*, (b) *Nab2^ex3^* larvae. Inset black boxes show high magnification views of dendritic arbors. (c) Schematic depicting measured dendritic parameters using Matlab TREES toolbox and custom scripts. (d) Balloon plot depicting 10 measurements of the *Nab2^ex3^* dendritic arbor. Heat map shows change percent changes in *Nab2^ex3^* vs *control*.

**Fig. 5.**
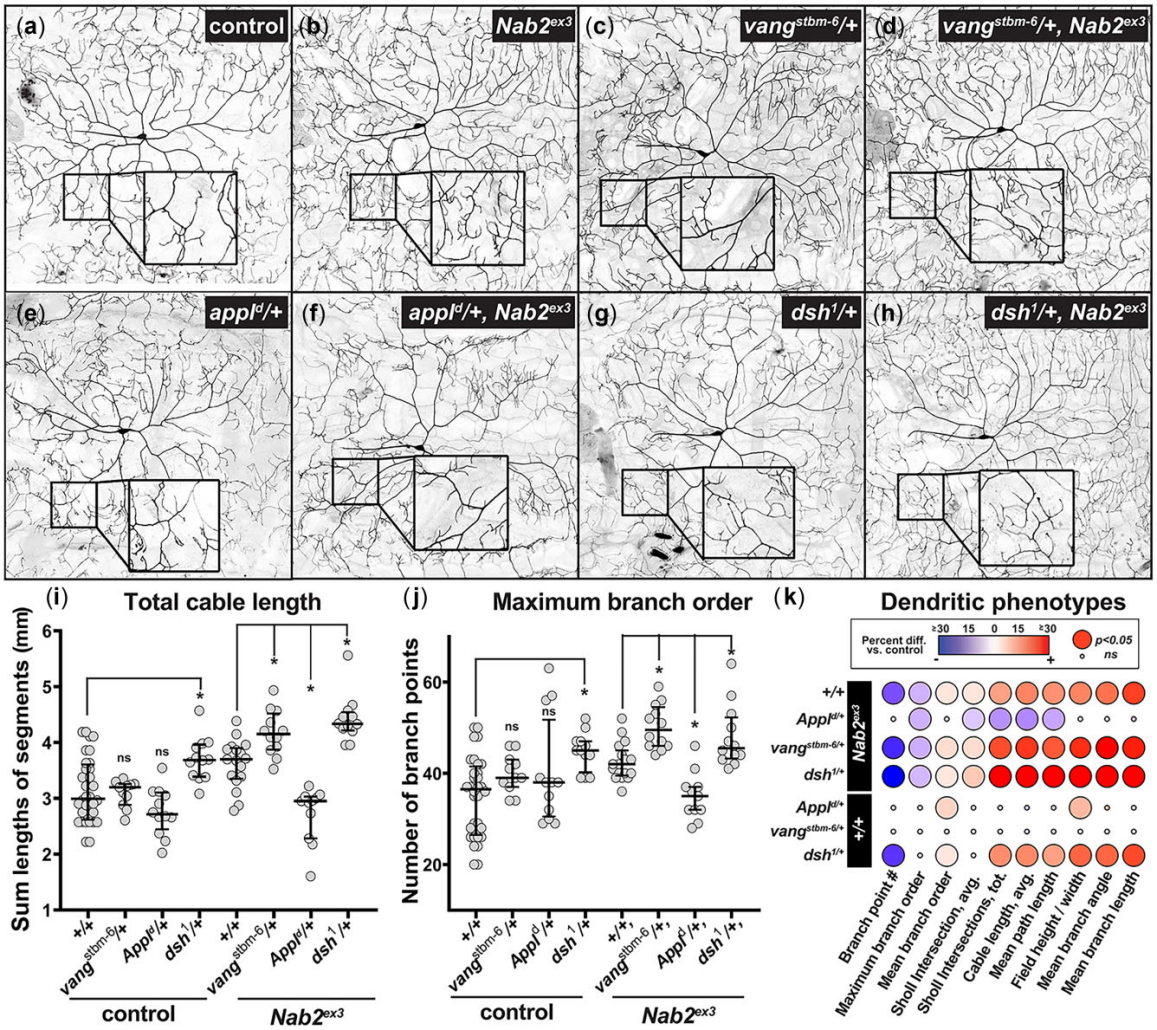
PCP components dominantly modify Nab2 dendritic phenotypes. Inverted intensity images of *Drosophila* class IV ddaC neurons from (a) *control +/+* or (b) *Nab2^ex3^* larvae alone, or in combination with (c, d) *Vang^stbm6^/+*, (e, f) *Appl^d^/+*, (g, h) *dsh^1^/+*. Inset black boxes show high magnification views of dendritic arbors. i, j) Quantification of (i) total cable length and (j) maximum branch order in the indicated genotypes; errors bars represent median and upper/lower quartile, *P <* *0.05. k) Balloon plot analysis of 10 arbor parameters in the indicated genotypes. Heat map shows change percent changes in *Nab2^ex3^* vs *control*. Significance depicted by balloon size (large balloon* *=* P *<* *0.05, small balloon* *=* *ns). Significance is determined using Student’s *t*-test or ANOVA as indicated in figure legends. Error bars in (i) and (j) represent standard deviation. Significance scores indicated are *P ≤* *0.05, **P ≤* *0.01, and ***P ≤* *0.001.

The data above confirm that Nab2 and the PCP pathway are each required within ddaC neurons to control the extent of dendritic branching. To further assess whether modulation of PCP pathway activity affects this newly defined *Nab2* dendritic role, we exploited the Matlab TREES toolbox and custom scripts to simultaneously quantify multiple dendritic phenotypes in *Nab2^ex3^* homozygous larvae (Fig. 4c; Cuntz *et al.* 2010). This approach confirmed that *Nab2* loss elevates the total number of branches compared to control (Fig. 4, a, b, and d) but also revealed an extension of overall cable length (Fig. 4, a–c) indicative of increased total projections. These data match the increase in intersections observed among *Nab2^ex3^* ddaC cells observed using the Sholl technique (Fig. 3h and Supplementary Fig. 2b). A further breakdown of *Nab2^ex3^* branching patterns using TREES parameters shows an increase in maximum branch order (number of branch points along a given branch from soma to distal tip; Fig. 5, i and j) and coupled decrease in mean branch length (distance between consecutive branches; Fig. 4d). Thus, *Nab2^ex3^* ddaC arbors project and branch significantly more than control across multiple parameters (Fig. 4d). Due to the increased branching, *Nab2^ex3^* ddaC arbors exhibit reduced mean path length (−4%), smaller mean branch angles (−9%), and smaller mean branch lengths (−22%) compared to control (Fig. 4d). In view of the finding that *ppk>Nab2^RNAi^* phenocopies the effect of *Nab2^ex3^* homozygosity on ddaC arbors (see Fig. 3, c and g), these TREES data are consistent with a model in which Nab2 is required in ddaC neurons to limit dendrite projection and branching.

### PCP alleles exhibit compartment-specific modification of *Nab2* null dendritic phenotypes

Having established that Nab2 loss elicits a spectrum of ddaC branching and projection defects, we proceeded to test whether genetic reduction of PCP components could affect one or more of these parameters. Single copies of the *Vang^stbm6^* and *Appl^d^* alleles (i.e. as heterozygotes) each have no significant effects on ddaC arbors in an otherwise wild-type background, while *dsh^1^* heterozygosity results in increased branch points, Sholl intersections, and total cable length compared to controls (Fig. 5, a–j). When placed into the *Nab2^ex3^* background, single copies of *Vang^stbm6^* and *Appl^d^* alleles dominantly modify *Nab2^ex3^* phenotypes in opposite directions: *Vang^stbm6^* enhances the severity of *Nab2^ex3^* ddaC branching and length phenotypes while *Appl^d^* suppresses many of the same phenotypes (e.g. total cable length and maximum branch order; Fig. 5, d, f, and I, and j). While the *dsh^1^* allele enhances *Nab2^ex3^* phenotypes (Fig. 5, h–j), ddaC defects in *dsh^1^* heterozygotes suggest that this could be an additive effect. Intriguingly, Sholl analysis reveals that the *Vang^stbm6^* allele primarily increases complexity in *Nab2^ex3^* proximal arbors (Supplementary Fig. 2b), which are not normally affected by Nab2 loss (see Fig. 3h). By contrast, the *Appl^d^* allele has a strong suppressive effect primarily on distal *Nab2^ex3^* arbors (Supplementary Fig. 2b). Collectively, these genetic and quantitative data argue that Nab2 acts within ddaC neurons to restrict branching and projection of their dendrite arbors, and that Nab2 loss sensitizes the proximal arbors to reduced Vang expression and the distal arbors to reduced expression of Appl. Given that individual PCP proteins could act within signaling or receiving cells, these compartment-specific effects could reflect functional interactions between Nab2-Vang and Nab2-Appl *within* ddaC dendrites, or *between* ddaC dendrites and the cellular substrates over which they grow.

## Discussion

Here, we uncover a role for *Drosophila* Nab2, an evolutionarily conserved RBP with links to human inherited intellectual disability, in cell-autonomous restriction of dendrite branching and projection among ddaC body-wall sensory neurons. Loss of Nab2 increases dendrite branching and projection while overexpression of Nab2 has the opposite effect of restricting dendrite growth. Using proteomic data collected from *Nab2* null developing fly brains (Corgiat *et al.* 2021), we uncover an enrichment for PCP factors among proteins whose steady-state levels are affected by Nab2 loss and define a pattern of genetic interactions consistent with Nab2 regulating projection and branching of ddaC dendrites and also MB axons by a common PCP-linked effect. Cell type-specific RNAi indicates that Nab2 acts cell autonomously to guide axon and dendrite growth, implying a potential link between Nab2 and one or more PCP components within ddaC neurons and MB KCs. Intriguingly, alleles of 2 neuronal PCP genes, *Vang* and *Appl*, differentially affect proximal vs distal growth of *Nab2^ex3^* ddaC arbors and α vs β-lobe MB axon projection, suggesting spatially variable mechanisms linking Nab2 and the PCP pathway even within single cells. Collectively, these data establish Nab2 as a required inhibitor of dendrite growth and branching in ddaC neurons and demonstrate that Nab2 autonomously restricts axonal and dendritic growth through a PCP-sensitive mechanism that has the potential to be conserved across species.

RBPs shape axon and dendrite architecture by modulating post-transcriptional regulation of neuronal mRNAs, including their export from the nucleus and trafficking, stability, and translation in the cytoplasm (Ravanidis *et al.* 2018; Schieweck *et al.* 2021). Of note, the analysis presented here shows that the effects of Nab2 on dendritic morphology are exaggerated in distal regions relative to proximal regions closer to the nucleus (Fig. 3h;Supplementary Fig. 2, a and b). One explanation of this effect is that Nab2 controls expression of an mRNA (or mRNAs) encoding a factor that guides branching and projection of more distal dendrites. While neuronal Nab2 protein is primarily nuclear (Pak *et al.* 2011), the protein is also detected in cytoplasmic messenger ribonucleoprotein granules and has a proposed role in translational repression in conjunction with the Fragile-X mental retardation protein homolog Fmr1 (Bienkowski *et al.* 2017), suggesting that cytoplasmic Nab2 may inhibit translation of mRNAs that traffic to distal dendrites and encode proteins that limit branching and projection. Core PCP proteins localize to membranes at distal tips of some *Drosophila* neuronal growth cones (e.g. Reynaud *et al.* 2015; Misra *et al.* 2016) and multiple *Drosophila* Wnt/PCP proteins act autonomously in ddaC cells to control dendritic growth [e.g. *fz2* in this study and see Matsubara *et al.* (2011)]. Considering these observations, Nab2 might regulate trafficking, translation, or turnover of one or more mRNAs that encode PCP components or regulators. Molecular identification of Nab2-bound mRNAs in the nuclei and cytoplasm of ddaC cells (e.g. by RIP-seq) would be required to test this hypothesis and to determine whether any candidate target RNAs encode PCP regulatory proteins.

As noted above, tissue- and compartment-specific genetic interactions between *Nab2* and PCP alleles imply that Nab2 loss sensitizes axons and dendrites to PCP gene dosage by different underlying mechanisms, including those that vary between cytoplasmic compartments of the same cell. For example, *Vang^sbm6^* heterozygosity selectively suppresses only *Nab2^ex3^* MB α-lobe defects, with no effect on β-lobe morphology. MB development is proposed to rely on a lobe-specific PCP mechanism involving the formin DAAM (Dsh associated activator of morphogenesis) interacting with Wg/Wnt receptor Frizzled (Fz) in the α-lobes and with Vang in the β-lobes (Gombos *et al.* 2015). A similar type of mechanism could occur for the Nab2–PCP interaction, with Nab2 either regulating different mRNAs in α vs β lobes or regulating factors that themselves have lobe-specific roles e.g. DAAM or the Derailed-Wnt5 receptor ligand pair (Reynaud *et al.* 2015). The α-lobe-specific *Nab2-Vang* genetic interactions mirror Nab2 interactions with alleles of 2 other RBPs, *fmr1* and *Atx2* (Bienkowski *et al.* 2017; Rounds *et al*. 2022), establishing a precedent for distinct *Nab2* genetic interactions in α vs β-lobe axons. Significantly, Nab2 associates with Fmr1 in the neuronal cytoplasm (Bienkowski *et al.* 2017) and limits ddaC dendrite growth, in part through an interaction with the mRNA encoding the PCP effector and small GTPase Rac1 (Fanto *et al.* 2000; Lee *et al.* 2003). These data provide one potential link between Nab2-Fmr1 and PCP activity in MB and ddaC neurons.

Dominant suppression of *Nab2^ex3^* mutant MB defects by the *Vang^stbm6^* allele is the inverse of how this same allele affects *Nab2^ex3^* ddaC phenotypes. One explanation of this effect could be that PCP signals exchanged between MB axons and surrounding neuro-substrate differ from those exchanged between ddaC neurons and their surrounding body-wall substrate, which could invert *Nab2* genetic interactions between MB and ddaC systems. Another factor to consider is the nonautonomy of some PCP alleles; e.g. while *Vang^stbm6^* brains show defective α and β-axon development, projection paths of individual *Vang^stbm6^* axon tracts can be rescued by adjacent *Vang* wild-type cells, indicating that Wnt/PCP control of α and β-axon branching is not strictly cell-autonomous (Shimizu *et al.* 2011; Ng 2012). In contrast to *Vang* alleles, partial loss of Appl (*Appl^d^*) consistently suppresses both *Nab2^ex3^* dendritic and axonal phenotypes (Supplementary Fig. 3), which parallels the increase in Appl protein detected in brain proteomics in *Nab2* mutant brains (Fig. 1b, Supplementary Table 1). Appl acts as a downstream neuronal-specific effector of the PCP pathway (Soldano *et al.* 2013; Liu *et al.* 2021) and elevated Appl protein in response to Nab2 loss could be an indirect consequence of altered core PCP pathway activity or evidence of direct regulation of the *Appl* transcript.

In aggregate, these data reveal a pattern of genetic interactions between *Nab2* and PCP alleles and provide the first evidence that Nab2 is required for dendritic development. These interactions between Nab2 and PCP proteins in ddaC and MB cells could be cell-autonomous or reflect interactions between neurons and surrounding substrate. Changes in expression levels of core PCP proteins, such as Vang, detected in proteomic analysis suggest that *Vang* mRNA is a candidate target of post-transcriptional control by Nab2 both in axons and dendrites. Given that loss of the Nab2 ortholog in mice, *Zc3h14*, also alters levels of the Vangl2 PCP protein in the adult hippocampus, and that mutations in PCP genes including *Vangl2* are linked to intellectual disabilities, severe neural tube closure defects, and microencephaly in humans (e.g. Wang *et al.* 2019) dysregulation of the PCP signaling in neurons is one potential mechanism to explain axonal and dendritic phenotypes in *Zc3h14* mutant mice (Jones *et al.* 2021) and cognitive defects in human patients lacking ZC3H14 (Pak *et al.* 2011).

## Data availability

Proteomics data have been deposited to the ProteomeXchange Consortium via the PRIDE partner repository with the dataset identifier PXD022984. All remaining data are contained within the article.

Supplemental material is available at *G3* online.

## Supplementary Material

jkac100_Figure_S1

jkac100_Figure_S2

jkac100_Figure_S3

jkac100_Table_S1
