# Supplementary material for: The Nab2 RNA-binding protein patterns dendritic and axonal projections through a planar cell polarity-sensitive mechanism
Source: G3 (Bethesda). 2022 Apr 26;12(6):jkac100. doi: 10.1093/g3journal/jkac100 (PMC9157165; doi:10.1093/g3journal/jkac100)
Supplement: jkac100_Figure_S1 [file jkac100_figure_s1.docx]

**Supplemental Figure 1: Variance in mushroom body (MB) morphological defects with PCP modifying alleles.** Confocal images of Fasciclin II (FasII) antibody staining of 48-72hr after pupal formation brain show maximum intensity Z-stack projections (projection) and single transverse section (single section) to highlight midline crossing of β-lobe axons. Dominant modification of *Nab2^ex3^* by single copy alleles of *Vang^stbm-6^, Appl^d^,* and *dsh^1^* is variable, with some examples shown here. Typical phenotypes are shown in **Figure 2 G,H,K,L,O,P**.
