# Supplementary material for: The Nab2 RNA-binding protein patterns dendritic and axonal projections through a planar cell polarity-sensitive mechanism
Source: G3 (Bethesda). 2022 Apr 26;12(6):jkac100. doi: 10.1093/g3journal/jkac100 (PMC9157165; doi:10.1093/g3journal/jkac100)
Supplement: jkac100_Figure_S2 [file jkac100_figure_s2.docx]

**Supplemental Figure 2: Proximal-distal effect on dendritic arbor complexity.** (A) Diagram depicting the concentric rings used to perform Sholl analysis overlaid on the dendritic arbor of a neuron. The half of the rings proximal to the soma labeled in **blue**; the half of the rings distal to the soma labeled in **red**. (B) Quantification of branching complexity by Sholl analysis using total Sholl intersections split across the proximal half, distal half, or full dendritic arbor. (B) Proximal-distal effect on total Sholl intersections of *control* compared to single copies of *Vang^stbm-6^*, *Appl^d^*, and *dsh^1^*; and of *Nab2^ex3^* compared to single copies of *Vang^stbm-6^*, *Appl^d^*, and *dsh^1^* in the background of *Nab2^ex3^*. Bars represent median and upper/lower quartile, * indicates *p*<0.05, **** indicates *p*<0.001. Control *n*=32, *Nab2^ex3^ n*=17.
