# Supplementary material for: The Nab2 RNA-binding protein patterns dendritic and axonal projections through a planar cell polarity-sensitive mechanism
Source: G3 (Bethesda). 2022 Apr 26;12(6):jkac100. doi: 10.1093/g3journal/jkac100 (PMC9157165; doi:10.1093/g3journal/jkac100)
Supplement: jkac100_Figure_S3 [file jkac100_figure_s3.docx]

**Supplemental Figure 3: Overview of dominant modification of *Nab2^ex3^* phenotypes by PCP component alleles.** Balloon plot depicting ten measurements of the ddaC neuron dendritic phenotypes and six measurements of the MB axon phenotypes. Change is shown as percent difference vs *control*; percent change ranges from increased in red to decreased in blue; significance depicted by balloon size (large balloon = *p*<0.05, small balloon = ns). The top four rows represent alleles in the *Nab2^ex3^* background; the bottom three rows represent alleles in *control* background.
